# Supplementary material for: Allelic Expression Imbalance Analysis Identified YAP1 Amplification in p53- Dependent Osteosarcoma
Source: Cancers (Basel). 2021 Mar 18;13(6):1364. doi: 10.3390/cancers13061364 (PMC8002920; doi:10.3390/cancers13061364)
Supplement: Supplementary file 1 [file cancers-13-01364-s001.pdf]

cancers MDPI

Supplementary Materials: Allelic Expression Imbalance Analysis Identified *YAP1* Amplification in p53- Dependent Osteosarcoma

Guanglin Niu, Agnieszka Bak, Melanie Manyet, Yue Zhang, Hubert Pausch, Tatiana Flisikowska, Angelika E. Schnieke and Krzysztof Flisikowski

Table S1. List of primers used in the study.

Primers	Sequence(5′---3′)	Application
YAP1_1F	TGATGAATTCGGCCTCAGGT	qpcr
YAP1_1R	GCGCTTTGACTGATTCTCTGG	
YAP1_2F	CGTCCGAGGCAAGTTTCTGT	pcr
YAP1_2R	GAAAAACAAATCTCGGCCCC	
Wrap53_1F	TGCAGAAGAAACGAACAAGCC	pcr
Wrap53_1R	TGGGGGCAGGTTATAAATCCG	
TP53INP1_1F	GTGAAGTCAATACTTCTTCC	pcr
TP53INP1_1R	AATCACTTGGGTCAGCTAGGC	
p14_1F	CGTGCTGTTGCTAGTGACGA	pcr
p14_1R	AGGCGTCTCGCACGTCTA	
p16_1F	AACGCACCGAACCGTTAC	pcr
p16_1R	AGGACCACCAAAGTGTCC	
Rb1_1F	CCACCGCAGCCTGAGGAGGA	pcr
Rb1_1R	CCACAGATGAAACCTTCTCC	
TP63_1F	GACCCTTACATCCAGCGGTT	pcr
TP63_1R	CCTGCATGCGAATACAGTCC	
TP73_1F	TCCACCTTCGACACCATGTC	pcr
TP73_1R	TGCTCCGCCTTCTTGTAGAT	
GAPDH_1F	TTCACGACCATGGAGAAGGC	pcr, qpcr
GAPDH_1R	GGTTCACGCCCATCACAAAC	
p16_MF1	AGTAGTGTTTTTAGGGGTGT	methylation
p16_MR1	CCATACTACTCCAAATAACTCTC	
p16_MS1	GGAAGGAGGGATTTATTG	
Rb1_MF1	TGGTTAAATTTAGTTTTTAA	methylation
Rb1_MR1	AAAAACCCCAACCTAAAAAT	
Rb1_MS1	TATTTAAGTTTGGAGGG	

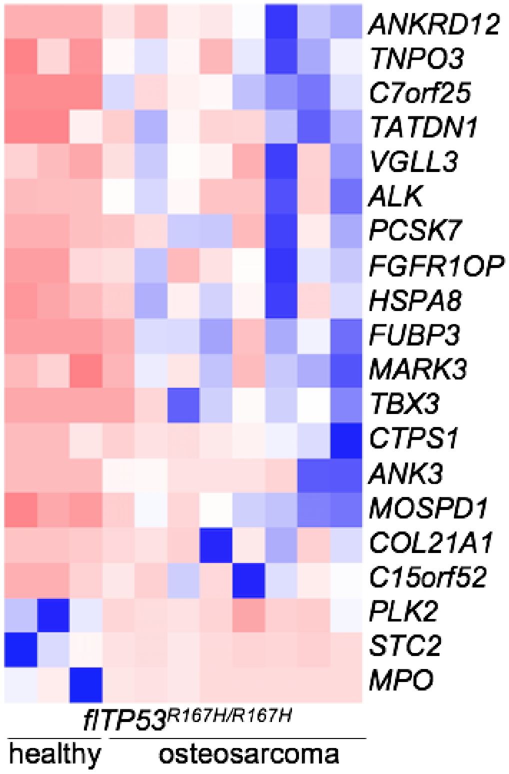

Figure S1. Heatmap of the top 20 differentially expressed genes in OS and partially matched healthy bone samples from fl*TP53*$^{R167H/R167H}$ pigs.

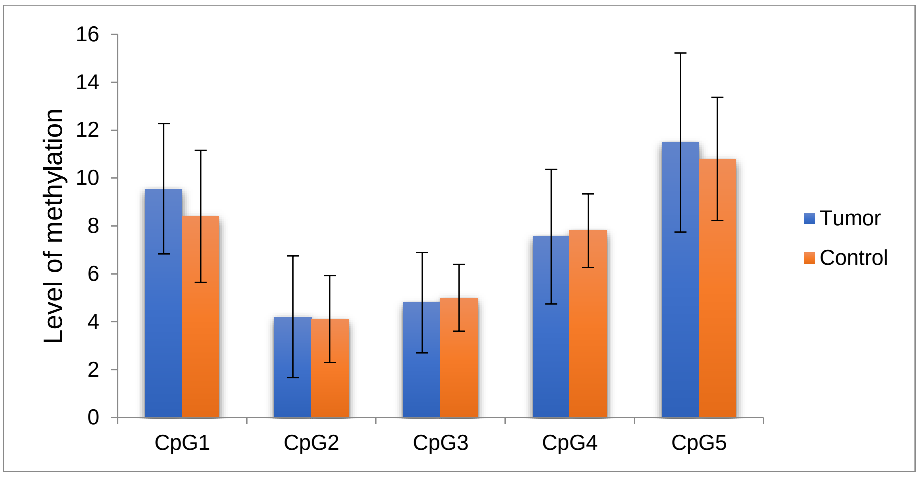

Figure S2. DNA Methylation of the BIRC3 promoter in OS and matched healthy control tissues.

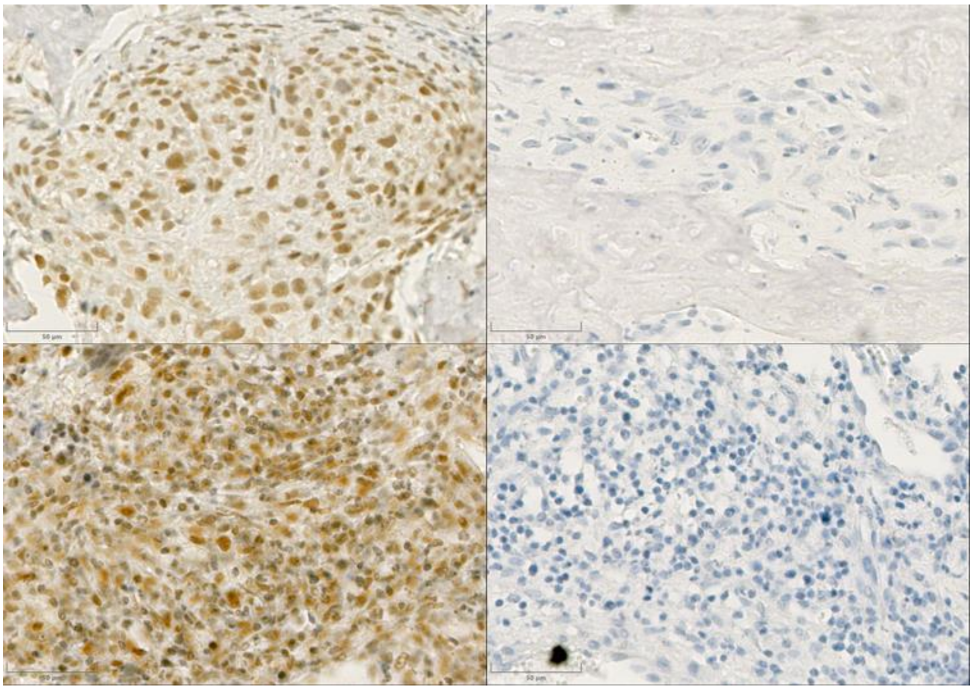

Figure S3. Immunostaining of YAP1 in OS samples.

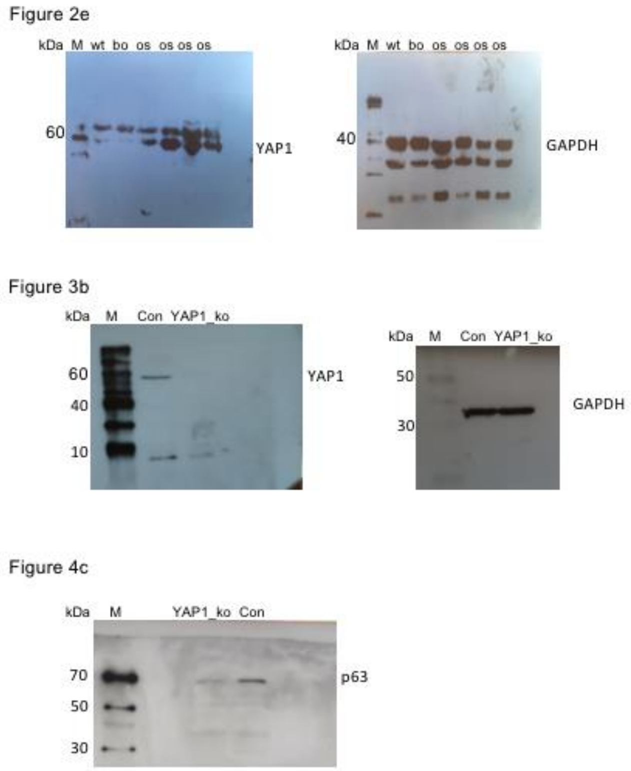

Figure S4. Unprocessed western blots.
